# The Use of Essential Oils and Their Isolated Compounds for the Treatment of Oral Candidiasis: A Literature Review

**DOI:** 10.1155/2021/1059274

**Published:** 2021-01-07

**Authors:** Elba dos Santos Ferreira, Pedro Luiz Rosalen, Bruna Benso, Janaina de Cássia Orlandi Sardi, Carina Denny, Simone Alves de Sousa, Felipe Queiroga Sarmento Guerra, Edeltrudes de Oliveira Lima, Irlan Almeida Freires, Ricardo Dias de Castro

**Affiliations:** ^1^Health Sciences Center, Federal University of Paraíba, João Pessoa 58051-970, PB, Brazil; ^2^Federal University of Alfenas, Alfenas, MG, Brazil; ^3^Department of Physiological Sciences, Piracicaba Dental School, University of Campinas, Piracicaba, SP, Brazil; ^4^Federal University of Mato Grosso do Sul, Faculty of Pharmaceutical Sciences, Pioneiros 79070-900, MS, Brazil; ^5^Department of Clinical and Social Dentistry, Federal University of Paraíba, Campus I, João Pessoa, PB, Brazil; ^6^Pharmaceutical Sciences Department, Health Sciences Center, Federal University of Paraíba, Campus I, João Pessoa, PB, Brazil; ^7^Department of Clinical and Social Dentistry, Health Sciences Center, Federal University of Paraíba, João Pessoa, PB, Brazil

## Abstract

In this literature review, we present the main scientific findings on the antifungal activity of essential oils (EOs) applicable for a new drug formulation to treat oral candidiasis. Seven literature databases were systematically searched for eligible in vitro and clinical trials. Selected articles were screened for biological activity, botanical species, phytochemical composition, study design, and methodological quality. A total of 26 articles were included in the review, of which 21 were in vitro studies and 5 clinical trials. The most promising EOs were obtained from *Allium tubeorosum*, *Cinnamomum cassia*, *Cinnamomum zeylanicum*, and *Coriandrum sativum* L. Among the phytochemicals, citral and thymol were the most active. Clinical trials indicated that the EOs from *Pelargonium graveolens* and *Zataria multiflora* are potentially effective to treat oral candidiasis. Further nonclinical and clinical studies with these EO are warranted to determine their potential use and safety for the treatment of oral candidiasis.

## 1. Introduction

Oral candidiasis is an infection caused by *Candida* spp. which manifests clinically as erythematous, ulcerated, sensitive white lesions, with soft consistency and easy removal, commonly affecting the palate, oral mucosa, tongue, or oropharynx [1]. Due to the opportunistic pathogenicity of yeasts, oral candidiasis is more prevalent in immunocompromised individuals [2].

Yeast colonization of complete dentures occurs mainly due to the strong adhesion of yeast cells to acrylic resin of base materials [1]. Direct adhesion of yeasts onto dental surfaces is a critical pathogenic factor for the onset of dental stomatitis. Yeast cells can co-aggregate with various bacterial species from the oral plaque and integrated into a robust biofilm pellicle on the surface of dentures. Oral biofilms can be considered microbial reservoirs and significantly affect the oral and systemic health of denture users [1, 3].

Polyene and azole drugs, such as nystatin and miconazole, respectively, have been commonly prescribed for the treatment of oral candidiasis [2]. However, recent years have seen failures in antifungal therapy due to increasing microbial resistance rates and high drug toxicity, which have altogether contributed to rise the prevalence of morbidity and mortality indicators related to fungal infections [4].

This scenario has encouraged the search for novel substances capable of controlling and treating yeast infections while having low toxicity to the host. Some naturally occurring products are considered an important source of new molecules with biological properties, displaying antifungal efficacy comparable or stronger than that of drugs currently available for clinical use. Essential oils (EOs) are a class of natural products with pharmacological properties, which include antimicrobial, antiseptic, anti-inflammatory, and antioxidant activities [5–9]. These compounds are described as a mixture of volatile constituents produced as secondary metabolites by aromatic plants. With the chemical characteristic of lipophilicity, EOs have the ability to interact with fungal cell membranes and lipid structures. Among their mechanisms of action, EO can disrupt the activity of enzymes involved in ergosterol synthesis or complex with membrane ergosterol, thereby creating pores in the membrane and disrupting permeability [10–12]. In addition, EO can affect cell wall biosynthesis, interfere with protein synthesis or cell division, and stimulate the production of reactive oxygen species, causing growth inhibition or cell death [13, 14]. In this literature review, we present the main scientific findings on the antifungal activity of EO and their isolated phytochemicals on *Candida* spp. commonly responsible for oral infections. In vitro and clinical (controlled clinical trials in humans) studies were selected and are further discussed in this review.

## 2. Materials and Methods

### 2.1. Study Question

This literature review was conducted to address the specific question: “Is there scientific evidence to support the use of EO and/or their isolated constituents for the treatment of oral candidiasis or to warrant further nonclinical and clinical research?”

### 2.2. Search Strategy and Study Selection

The PRISMA guidelines (Transparent Reporting of Systematic Reviews and Meta-Analyses) [15] were followed. Seven databases were systematically searched for studies of experimental oral candidiasis and randomized controlled clinical trials published up to 1 March 2020 (Table 1).

### 2.3. Eligibility Criteria

A systematic screening of the articles was performed by two independent examiners according to the following inclusion criteria:

1. Biological activity: clinical effects of an EO-containing formulation on denture stomatitis or oral candidiasis in in vitro or clinical trials. Primary outcome of interest: antifungal activity of the EO and/or isolated constituent based on their MIC (minimum inhibitory concentration). Secondary outcome of interest: reduction in CFUs (colony-forming units) after treatment with the EO-containing formulation leading to remission or cure of infection. Tertiary outcome of interest: cure or reduction in the size and number of erythematous lesions upon treatment with the EO-containing formulation.
2. Plant material and chemical elucidation: chemically characterized EO and/or their isolated constituents from aromatic plants.
3. Study design: in vitro studies and phases I, II, III or IV clinical trials. Sample size and study power (at least 80%) should be adequate to determine accurate statistical inferences.
4. Methodological quality: accuracy of methods and outcomes; internal and external validity; for clinical trials—high quality standards.
5. Language: articles written in English, Spanish, or Portuguese. Examiners agreed that in cases of inconsistence, the final verdict on which articles should be included would be reached by consensus.

### 2.4. Data Analysis

For in vitro studies, a range of MIC values was used as a parameter to determine the extent of antifungal activity for interstudy comparisons (adapted from [16]). The established scoring criteria for MIC values are shown in Table 2.

Randomized controlled trials of herbal interventions were analyzed based on the CONSORT guidelines [17]. The Jadad scale [18] was used to check study validity and methodological quality (randomization, blinding, and loss of follow-up). Based on these requirements, clinical studies were assigned scores ranging from 0 to 5, in which a score <3 was indicative of poor quality.

## 3. Results

### 3.1. Search Strategy

**Figure 1:**
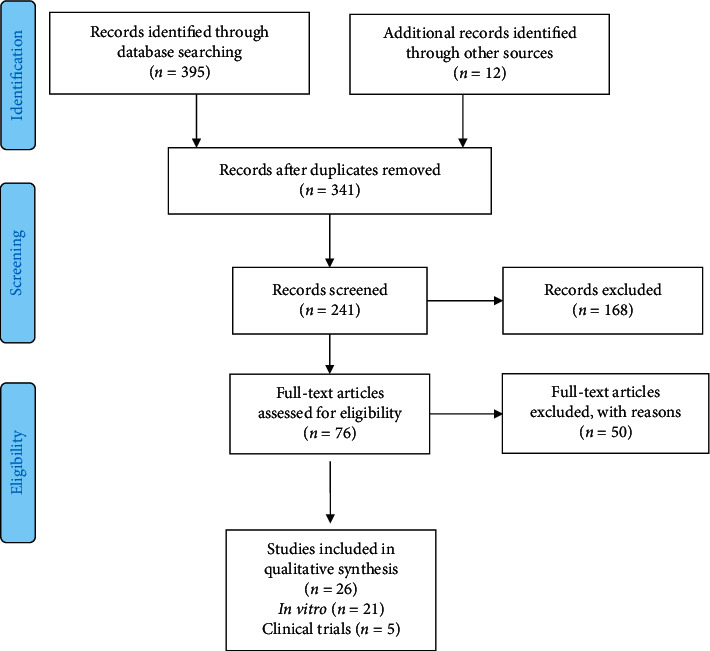
Flow diagram of the search strategy comprising the identification of potentially relevant material, preliminary screening (based on PRISMA guidelines) (main categories by which the articles were excluded from the study).

Using a previously defined strategy, bibliographic searches were carried out using specific keyword combinations. A total of 395 articles were retrieved, of which 26 were considered eligible and included in the final review (Figure 1). Twenty-one studies with in vitro design and five clinical trials were included and are further discussed.

### 3.2. In Vitro Antifungal Activity

The antifungal activity of thirty-one EO and four phytochemicals against *Candida* spp. strains (clinical isolates and reference strains) was analyzed. As shown in Tables 3 and 4, the most promising EOs were obtained from *Allium tuberosum*, *Cinnamomum cassia*, *Cinnamomum zeylanicum*, and *Coriandrum sativum*. Citral and Thymol were the most active isolated constituents, with MIC values lower than 100 *μ*g/mL, indicating very strong antifungal activity (Table 5)[.

### 3.3. Randomized Clinical Trials

#### 3.3.1. Effects of Intervention

**Figure 2:**
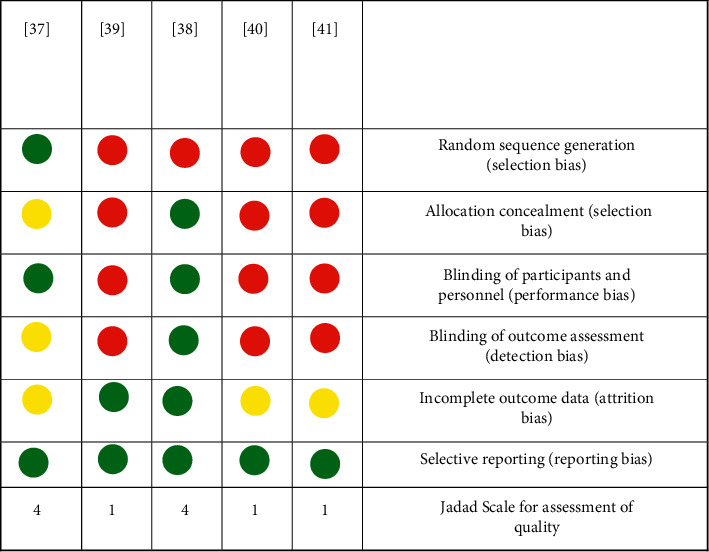
Risk-of-bias summary of the clinical trials included in this literature review. Red (−) stands for high risk of bias, green (+) stands for low risk of bias, and yellow (?) stands for unclear risk of bias. Overall, the studies are compliant with the CONSORT guidelines for clinical trials of herbal interventions, showing a low risk of bias.

According to the pre-established criteria, five clinical studies were included in this review: *Pelargonium graveolens*, *Zataria multiflora*, and *Melaleuca alternifolia* in three formulations. The main methodological characteristics and outcomes of selected studies are shown in Table 6 and Figure 2. An experimental gel containing *Pelargonium graveolens* EO healed completely (34%) or partially (56%) patients with prosthetic stomatitis as compared to those who received only the gel with a placebo. In addition, the gel was effective in reducing the fungal load as well as in decreasing erythema in patients with prosthetic stomatitis as compared to those treated with the placebo. Another experimental gel containing *Zataria multiflora* EO was also effective in reducing the fungal load in participants' saliva and denture samples as well as *n* reducing local inflammation.

## 4. Discussion

The main antimicrobial mechanisms of EO and their constituents are associated with their ability to increase cell membrane permeability due to lipophilicity of their molecules, resulting in extravasation of ions and cellular contents and cell lysis [39–41]. In this review, the selected data suggest that some EO and phytochemicals are promising for the treatment of oral candidiasis and warrant further nonclinical, clinical, and toxicological investigation for pharmaceutical purposes [42–44].

Next, a brief summary of the most active EO and isolated compounds will be presented based on in vitro and clinical studies. Information on ethnopharmacological knowledge, biological properties, and chemical composition is further discussed.

### 4.1. Essential Oils and Phytochemicals with Promising Antifungal Activity against *Candida* spp

The *Allium* genus, which belongs to the Amaryllidaceae family, contains approximately 700 species of plants, such as *Allium cepa* (onion), *Allium sativum* (garlic), *Allium schoenoprasum* (chives), and *Allium tuberosum* (garlic chives). All are important due to their commercial character and nutritional value [45]. *Allium tuberosum* is a perennial plant that grows in many countries in Asia and whose aerial parts are edible green vegetables common to the Chinese. *A. tuberosum* has an odor similar to the smell of garlic and other *Allium* plants due to the presence of sulfur-containing compounds [46].

Several pharmacological activities are attributed to this species, including antidiabetic and hepatoprotective [47], antiparasitic [48], antibacterial [49], and antifungal activities against fungi of the *Aspergillus* genus [50]. This species has been reported to have strong antifungal activity against *Candida parapsilosis* isolates and inhibitory effects on biofilm formation [19].

*Cinnamomum cassia*, popularly known as China cinnamon, is an herb belonging to the Lauraceae family, occurring in several countries such as India, China, Uganda, Vietnam, Bangladesh, and Pakistan. It is intensely aromatic, with a sweet taste and bitter touch. Its peels have been used in different ways, either as a flavoring in various Asian cuisines or in traditional medicine for the treatment of diabetes mellitus and peptic ulcer [51]. The major compound of *C. cassia* is cinnamaldehyde (75–90%). Other phytoconstituents, present in trace amounts, include eugenol, benzoic acid, cinnamic acid, salicylic acid, cinnamyl alcohol, and their corresponding esters and aldehydes [52].

*C. cassia* has been shown to have anti-inflammatory, antioxidant, anticancer, antipyretic, antiangiogenic, larvicidal, and antifungal properties [53]. *C. cassia* was reportedly active against four *Candida* spp. strains, namely, *C. albicans* and *C. tropicalis*, *C. glabrata*, and *C. krusei*, as well as against *Aspergillus*, *Fusarium*, and three dermatophyte isolates (*Microsporum gypseum*, *Trichophyton rubrum*, and *T. mentagraphytes*). *C. Cassia* EO was effective in reducing the number of pseudohyphae in *C. albicans* cultures, which is considered an important virulence factor [54]. Mouse models and in vitro assays have also proved the antiproliferative activity of *C. cassia* EO against oral candidiasis (*C. albicans* infection). Cinnamaldehyde was reported as the main compound responsible for the antifungal effects observed in *C. cassia* EO [55].

*Cinnamomum zeylanicum*, popularly known as cinnamon, is a very common spice that has been used by different cultures around the world for several centuries. It is obtained from the bark and leaves of trees of the genus *Cinnamomum*, a perennial tropical plant that has two main varieties, namely, *Cinnamomum zeylanicum* and *Cinnamomum cassia.* In addition to its culinary uses, in native Ayurvedic medicine, cinnamon is used as an alternative to treat respiratory, digestive, and gynecological diseases [56]. Four of the main components of the EO obtained from *C. zeylanicum* bark are trans-cinnamaldehyde, cinnamaldehyde, eugenol, and linalool, which represent 82.5% of the total EO composition [57]. In vitro and in vivo studies in animals and humans have shown important biological activities attributed to *C. zeylanicum* EO, such as anti-inflammatory, antimicrobial, reduction of cardiovascular diseases, and increase of cognitive function [58]. Some studies reported that *C. zeylanicum* EO has antifungal activity against *Candida* spp. most likely by disrupting yeast cell wall [14, 24, 25], which suggests that this EO may be a promising candidate for the treatment of oral candidiasis.

*Coriandrum sativum* L. is a small plant belonging to the *Apiaceae* family, popularly known as coriander. Coriander leaves and seeds are widely used in folk medicine as a cholesterol-lowering agent, digestive stimulant, and antihypertensive [11], in addition to its use as a spice in food preparation. The main components present in *C. sativum* EO are linalool (55.09%), *α*-pinene (7.49%), 2,6-octadien-1-ol, 3,7-dimethyl-acetate, geraniol (4.83%), 3-cyclohexene-1-methanol, *α*, *α*, 4-trimethyl- (4.72%), hexadecanoic acid (2.65%), acid tetradecanoic (2.49%), 2-*α*-pinene (2.39%), citronellyl acetate (1.77%), and undecanal (1.29%) [59]. Pharmaceutical formulations containing *C. sativum* also revealed antibacterial [60], antioxidant [61], hepatoprotective, and anticonvulsant properties. *C. sativum* EO also showed strong antifungal effects against *Candida* spp. strains [16].

Citral (3,7-dimethyl-2-6-octadienal) is a racemic mixture composed of geranial (trans-citral, citral A) and neral (cis-citral, citral B) isomers, which are acyclic and monounsaturated aldehydes naturally occurring in many citric fruits, as well as in other herbs or spices [62]. Citral has become a raw material of great importance due to its characteristic lemon aroma and has been used as a flavoring ingredient in the food, perfumery and cosmetic industries [63]. Citral showed fungicidal activity against *Candida* spp. strains isolated from denture wearers after 2 hours of exposure and caused major morphological changes [34]. Leite et al. [64] demonstrated a strong antifungal activity of citral against *C. albicans* strains via mechanisms other than cell wall biosynthesis or ergosterol complexation. Thus, citral can be considered a promising candidate for the development of novel antifungal leads.

Thymol is a monoterpene found in essential oils extracted from plants belonging to the Lamiaceae family such as the genera *Thymus*, *Ocimum*, *Origanum*, *Satureja*, *Thymbra*, and *Monarda* [65–67]. This molecule is a phytoconstituent with several biological activities described, including anti-inflammatory and antinociceptive [68], local anesthetic [69], and antifungal and antibacterial [70] activities. Thymol has been reported to have strong antifungal activity against strains of the *Candida* genus, acting on the fungal cell membrane and producing a synergistic effect when used with nystatin to inhibit the growth of these strains [36].

### 4.2. Clinical Studies of Essential Oils for the Treatment of Oral Candidiasis

While numerous studies are carried out to determine the antifungal activity of EO in vitro, only a few formulations reach the clinical stage and even less become a commercial product. As seen in this review, few clinical trials have been carried out to test experimental formulations containing EO and/or isolated constituents against oral candidiasis. Currently, the most common formulations for the treatment of oral candidiasis are for external use, such as oral solutions, gels, and creams, which are normally safe [71].

Sabzghabaee et al. [37] evaluated the clinical efficacy of a gel containing *Pelargonium graveolens* EO for the treatment of prosthetic stomatitis. This study presented a low risk of bias for aspects related to randomization and blinding and showed high methodological quality according to Jadad's scale [18]. Another clinical study, conducted by Amanlou et al. [38], showed that *Zataria multiflora* EO is also effective to treat prosthetic stomatitis. Denture wearers applied the gel containing 0.1% of *Z*. *multiflora* EO four times a day for two weeks. The presence of erythema on the palate surface of participants was significantly reduced as well as CFU counts of yeast strains. Although limitations related to randomization were observed in the study by Amanlou et al. [38], it showed a low risk of bias, which suggests that *Z*. *multiflora* EO may be a favorable therapeutic alternative for the treatment of prosthetic stomatitis.

Despite the favorable outcomes of EO on oral candidiasis and prosthetic stomatitis reported by the authors of the studies selected in this review, only the studies with *P*. *graveolens* (popular names: fragrant-leaf geranium-Port., rose geranium-Engl., and geranium-Span.) and *Z*. *multiflora* (popular name: thyme of shiraz-Engl.) met high methodological quality standards. Further research should consider the chemical standardization of these EO and the adoption of appropriate methodological strategies for further clinical testing.

This literature review shows that the most promising EOs were obtained from *Allium tubeorosum*, *Cinnamomum cassia*, *Cinnamomum zeylanicum*, and *Coriandrum sativum* L. Among the phytochemicals, the citral and the thymol were the most active. The clinical trials selected in this review provided evidence that the EO from *Pelargonium graveolens* and *Zataria multiflora* are potentially effective to treat oral candidiasis. Further nonclinical and clinical studies with these EO are warranted to determine their potential use and safety for the treatment of oral candidiasis.

## Figures and Tables

**Table 1:** Search strategy and bibliographic databases used to retrieve the articles for this systematic review.

Primary bibliographic sources	Search strategy (descriptors and combinations with Boolean operators)
SciVerse Scopus (since 1995)	(i) (Essential oil AND oral candidiasis)
	(ii) (Oils, volatile, OR essential oil) AND (oral candidiasis)
	(iii) (Oils, volatile, OR essential oil) AND (denture stomatitis)

Web of Science (since 1990)	Filters; article or review and language
	(i) (Essential oil AND oral candidiasis)
	(ii) (Oils, volatile, OR essential oil) AND (oral candidiasis)
	(iii) (Oils, volatile OR essential oil) AND (denture stomatitis)

Medline via Pubmed (since 1966)	(i) (Essential oil AND oral candidiasis)
	(ii) (Oils, volatile OR essential oil) AND (oral candidiasis)
	(iii) (Oils, volatile OR essential oil) AND (denture stomatitis)

SciELO (Scientific Electronic Library Online) (since 1998), LILACS (Latin American and Caribbean Health sciences Literature) (since 1982), and Cochrane Library	(i) (Essential oil AND oral candidiasis)
	(ii) (Oils, volatile OR essential oil) AND (oral candidiasis)
	(iii) (Oils, volatile OR essential oil) AND (denture stomatitis)
	(iv) (Aceite esencial y candidiasis oral)
	(v) (EO e candidíase oral)
	(vi) (EO e estomatite protética)

Google Scholar	(i) Manual searches according to the reference lists of the articles

Search strategy and bibliographic databases and keywords.

**Table 2:** Established parameters based on minimum inhibitory concentrations of essential oils or related chemical constituents.

MIC range (*μ*g/mL)	Intensity of antifungal activity	Score
<100	Very strong activity	++++
101–500	Strong activity	+++
501–1000	Moderate activity	++
1000–2000	Weak activity	+
>2001	No activity	−

Source: adapted from Freires et al. [16].

**Table 3:** In vitro antifungal activity of essential oils against *Candida albicans* strains.

Plant species	Source	Microorganism	^MIC^50% (*μ*g/mL)	Score MIC	Ref
*Achillea millefolium*	Leaves	*C. albicans* clinical strain	625	++	[3]
*Allium tuberosum* Rottl. ex Spreng	Commercial Source	*C. albicans* CBS 562	500	+++	[19]
		*C. albicans* clinical strain	500	+++	
*Anethum graveolens*	Seeds	*C*. *albicans* ATCC (62342)	>2001	−	[20]
		*Candida albicans* clinical strain	>2001	−	
*Bursera morelensis*	Leaves	*C. albicans* clinical strain	125	+++	[21]
*Cinnamomum burmannii*	Commercial source	*C. albicans ATCC* 28366	1000	++	[22]
*Cinnamomum cassia*	Bark	*C. albicans* ATCC 76485	64	++++	[23]
		*C. albicans* C01-C11	64	++++	
*Cinnamomum zeylanicum*	Commercial source	*C. albicans* ATCC 76845	312.5	+++	[24]
*Cinnamomum zeylanicum*	Commercial source	*C. albicans* ATCC 76485	312.5	+++	[25]
		*C. albicans* ATCC 76645	312.5	+++	
		*C. albicans* clinical strain	625	++	
*Cinnamomum zeylanicum* Blume	Commercial source	*C. albicans* ATCC 40277	312.5	+++	[14]
		*C. albicans* clinical strain	312.5	+++	
*Cinnamomum zeylanicum* Blume	Commercial source	*C. albicans* CBS 562	250	+++	[26]
		*C. albicans* ATCC 60193	250	+++	
		*C. albicans* ATCC 90029	125	+++	
		*C. albicans* LM01	250	+++	
		*C. albicans* LM03	250	+++	
		*C. albicans* LM04	500	+++	
*Coriandrum sativum*	Commercial source	*C. albicans* CBS 562	15.0	++++	[19]
		*C. albicans* clinical strain 3 A5	31.0	++++	
*Coriandrum sativum L*.	Leaves	*C. albicans* CBS 562	15.6	++++	[27]
*Cuminum cyminum*	Seeds	*C. albicans* ATCC62342	>2001	−	[20]
		*C*. *albicans* clinical strain	>2001	−	
*Curcuma longa L.*	Rhizomes	*C. albicans* Clinical strain	625	++	[3]
*Laurus nobilis L.*	Commercial source	*C. albicans* ATCC 60193	250	+++	[28]
*Melaleuca alternifólia*	Commercial source	*C. albicans* ATCC 289065	>2001	−	[29]
		*C. albicans* ATCC 40277	>2001	−	
*Melaleuca alternifolia*	Commercial source	*C. albicans* ATCC 18804	1950	+	[30]
*Melaleuca alternifolia*	Commercial source	*C. albicans* clinical strain	>2001	−	[31]
*Pimpinella anisum*	Seeds	*C*. *albicans* ATCC 62342	>2001	−	[20]
		*Candida albicans* clinical strain	>2001	−	
*Ocotea odorifera*	Commercial source	*C. albicans* ATCC-90028	>2001	−	[24]
		*C. albicans* ATCC-76615	>2001	−	
		*C. albicans* ATCC-76645	>2001	−	
		*C. albicans* ATCC-76485	>2001	−	
		*C. albicans* clinical strain	>2001	−	
*Rosmarinus officinalis*	Commercial source	*C. albicans*, ATCC 289065	562,5	++	[29]
		*C. albicans* ATCC 40277	<2001	−	
*Rosmarinus officinalis* L.	Commercial source	*C. albicans* ATCC-90028	>2001	−	[24]
		*C. albicans* ATCC-76615	>2001	−	
		*C. albicans* ATCC-76645	>2001	−	
		*C. albicans* ATCC-76485	>2001	−	
		*C. albicans* clinical strain	>2001	−	
*Santolina chamaecyparissus*	Commercial source	*C. albicans* CBS 562	1000	++	[19]
		*C. albicans* clinical strain	>2001	−	
*Satureja hortensis L*.	Leaves	*C. albicans* Clinical strain	200	+++	[32]

nt (not tested); comparative MIC values (*μ*g/mL): (++++) <100; (+++) 100 to 500; (++) 501 to 1000; (+) >1001 to 2000; (−) >2001.

**Table 4:** In vitro antifungal activity of essential oils against non-*albicans Candida* strains.

Plant species	Source	Microorg.	*C. dubliniensis* ^1^		*C. glabrata* ^2^		*C. krusei* ^3^		*C. parapsilosis* ^4^		*C. tropicalis* ^5^		Ref.
			MIC_50%_ (*μ*g/mL)	Score MIC	MIC_50%_ (*μ*g/mL)	Score MIC	MIC_50%_ (*μ*g/mL)	Score MIC	MIC_50%_ (*μ*g/mL)	Score MIC	MIC_50%_ (*μ*g/mL)	Score MIC	
*Ageratum conyzoides* L	Leaves	ATCC 13803^5^	nt	−	nt	−	nt	−	nt	−	1000	++	[33]
		Clinical strain^5^	nt	−	nt	−	nt	−	nt	−	1000	++	

*Allium tuberosum* Rottl. Ex Spreng	Commercial source	CBS 604^4^	<2001	−	nt	−	nt	−	15	++++	nt	−	[19]
		CBS 7987^1^	Nt	−	nt	−	nt	−	nt	−	nt	−	
		*C*linical strain^3^	nt	−	nt	−	<2001	−	nt	−	nt	−	
		Clinical strain^4^	nt	−	nt	−	nt	−	15	−	nt	−	
		Clinical strain^1^	<2001	−	nt	−	nt	−	nt	−	nt	−	

*Anethum graveolens*	Seeds	Clinical strain^2,3,4^	nt	−	>2001	−	>2001	−	>2001	−	Nt	−	[20]

*Artemisia absinthum* L	Leaves	ATCC 13803^5^	nt	−	nt	−	nt	−	nt	−	500	+++	[33]
		Clinical strain^5^	nt	−	nt	−	nt	−	nt	−	500	+++	

*Artemisia camphorata* L	Leaves	ATCC 13803^5^	nt	−	nt	−	nt	−	nt	−	1000	++	[33]
		Clinical strain^5^	nt	−	nt	−	nt	−	nt	−	1000	++	

*Bidens sulphurea*	Leaves	ATCC 13803^5^	nt	−	nt	−	nt	−	nt	−	500	+++	[33]
		Clinical strain^5^	nt	−	nt	−	nt	−	nt	−	500	+++	

*Chenopodium ambrosioides* L	Leaves	ATCC 13803^5^	nt	−	nt	−	nt	−	nt	−	500	+++	[33]
		Clinical strain^5^	nt	−	nt	−	nt	−	nt	−	500	+++	

*Cinnamomum zeylanicum*	Commercial source	ATCC 13803^5^	nt	−	nt	−	nt	−	nt	−	625	++	[25]
		Clinical strain^5^	nt	−	nt	−	nt	−	nt	−	625	++	

*Cinnamomum zeylanicum* Blume	Commercial source	ATCC 40042^5^	nt	−	nt	−	nt	−	nt	−	312.5	+++	[14]
		Clinical strain^5^	nt	−	nt	−	nt	−	nt	−	312.5	+++	
		ATCC 40147^3^	nt	−	nt	−	312.5	+++	nt	−	nt	−	
		Clinical strain^3^	nt	−	nt	−	312.5	+++	nt	−	nt	−	

*Cinnamomum zeylanicum* Blume	Commercial source	ATCC 3413^3^	nt	−	nt	−	1000	++	nt	−	nt	−	[26]
		CBS 94^5^	nt	−	nt	−	nt	−	nt	−	25	+++	
		ATCC 750^5^	nt	−	nt	−	nt	−	nt	−	250	+++	
		Clinical strain^5^	nt	−	nt	−	nt	−	nt	−	250	+++	
		—	nt	−	nt	−	nt	−	nt	−	250	+++	
		—	nt	−	nt	−	nt	−	nt	−	625	++++	

*Citrus reticulata* Blanco	Leaves	ATCC 13803^5^	nt	−	nt	−	nt	−	nt	−	500	+++	[33]
		Clinical strain^5^	nt	−	nt	−	nt	−	nt	−	500	+++	

*Coreopsis lanceolata* L.	Leaves	ATCC 13803^5^	nt	−	nt	−	nt	−	nt	−	500	+++	[33]
		Clinical strain^5^	nt	−	nt	−	nt	−	nt	−	1000	++	

*Coriandrum sativum*	Commercial source	CBS 573^3^	nt	−	nt	−	15	++++	nt	−	nt	−	[19]
		CBS 604^4^	nt	−	nt	−	nt	−	125	+++	nt	−	
		CBS 7987^1^	7	++++	nt	−	nt	−	nt	−	nt	−	
		CBS 94^5^	nt	−	nt	−	nt	−	nt	−	125	+++	
		Clinical strain^3^	nt	−	nt	−	7	++++	nt	−	nt	−	
		Clinical strain^4^	nt	−	nt	−	nt	−	7	++++	nt	−	
		Clinical strain^1^	7	++++	nt	−	nt	−	nt	−	nt	−	
		Clinical strain^5^	nt	−	nt	−	nt	−	nt	−	63	++++	

*Coriandrum sativum* L.	Leaves	CBS 94^5^	nt	−	nt	−	nt	−	nt	−	312	++++	[27]
		CBS 573^3^	nt	−	nt	−	156	++++	nt	−	nt	−	
		CBS 7987^1^	31.2	++++	nt	−	nt	−	nt	−	nt	−	

*Cuminum cyminum*	Seeds	Clinical strain^2,3,4^	nt	−	>2001	−	>2001	−	>2001	−	Nt	−	[20]

*Foeniculum vulgare*	Leaves	Clinical strain^2,3,4^	nt	−	>2001	−	>2001	−	>2001	−	Nt	−	[20]

*Laurus nobilis* L.	Commercial source	ATCC 750^5^	nt	−	nt	−	nt	−	nt	−	250	+++	[28]
		ATCC 3413^3^	nt	−	nt	−	500	+++	nt	−	nt	−	
		CBS 94^5^	nt	−	nt	−	nt	−	nt	−	500	+++	
		CBS 573^3^	nt	−	nt	−	500	+++	nt	−	nt	−	
		—	nt	−	500	+++	nt	−	nt	−	nt	−	

*Lavandula officinalis* L.	Leaves	ATCC 13803^5^	nt	−	nt	−	nt	−	nt	−	500	+++	[33]
		Clinical strain^5^	nt	−	nt	−	nt	−	nt	−	500	+++	

*Melaleuca alternifólia*	Commercial source	ATCC 40147^3^	nt	−	nt	−	>2001	−	nt	−	nt	−	[29]
		ATCC 40042^5^	nt	−	nt	−	nt	−	nt	−	562.5	++	
		ATCC 13803^5^	nt	−	nt	−	nt	−	nt	−	>2001	−	

*Ocimum gratissimum* L	Leaves	ATCC 13803^5^	nt	−	nt	−	nt	−	nt	−	500	+++	[33]
		Clinical strain^5^	nt	−	nt	−	nt	−	nt	−	500	+++	

*Ocotea odorifera*	Commercial source	ATCC-13803^5^	nt	−	nt	−	nt	−	nt	−	<2000	−	[24]
		Clinical strain^5^	nt	−	nt	−	nt	−	nt	−	<2000	−	

*Pelargonium graveolens* L'H'er	Leaves	ATCC 13803^5^	nt	−	nt	−	nt	−	nt	−	125	+++	[33]
		Clinical strain^5^	nt	−	nt	−	nt	−	nt	−	125	+++	

*Pimpinella anisum*	Seeds	Clinical strain	nt	−	>2001	−	>2001	−	>2001	−	—	—	[20]

*Plectranthus neochilus* Schl	Leaves	ATCC 13803^5^	nt	−	nt	−	nt	−	nt	−	1000	++	[33]
		Clinical strain^5^	nt	−	nt	−	nt	−	nt	−	1000	++	

*Rosmarinus officinalis*	Commercial source	ATCC 40147^3^	nt	−	nt	−	1125	++	nt	−	nt	−	[29]
		ATCC 40042^5^	nt	−	nt	−	nt	−	nt	−	562.5	++	
		ATCC 13803^5^	nt	−	nt	−	nt	−	nt	−	1125	+	

*Rosmarinus officinalis* L.	Commercial source	ATCC-13803^5^	nt	−	nt	−	nt	−	nt	−	<2001	−	[24]
		Clinical strain^5^	nt	−	nt	−	nt	−	nt	−	<2001	−	

*Santolina chamaecyparis sus*	Commercial source	CBS 573^3^	nt	−	nt	−	500	+++	nt	−	nt	−	[19]
		CBS 604^4^	nt	−	nt	−	nt	−	>2001	−	nt	−	
		CBS 7987^1^	63	++++	nt	−	nt	−	nt	−	nt	−	
		CBS 94^5^	nt	−	nt	−	nt	−	nt	−	>2001	−	
		Clinical strain^3^	500	+++	nt	−	nt	−	nt	−	nt	−	
		Clinical strain^4^	nt	−	nt	−	nt	−	500	+++	nt	−	
		Clinical strain^1^	nt	−	nt	−	nt	−	nt	−	nt	−	
		Clinical strain^5^	nt	−	nt	−	nt	−	nt	−	>2001	−	

*Syzigium aromaticum*	Leaves	ATCC 13803^5^	nt	−	nt	−	nt	−	nt	−	500	+++	[33]
		Clinical strain^5^	nt	−	nt	−	nt	−	nt	−	500	+++	

*Tagetes erecta* L.	Leaves	ATCC 13803^5^	nt	−	nt	−	nt	−	nt	−	500	+++	[33]
		Clinical strain^5^	nt	−	nt	−	nt	−	nt	−	500	+++	

*Tetradenia Riparia* (Hochst.) Codd	Leaves	ATCC 13803^5^	nt	−	nt	−	nt	−	nt	−	250	+++	[33]
		Clinical strain^5^	nt	−	nt	−	nt	−	nt	−	250	+++	

nt (not tested); comparative MIC values (*μ*g/mL): (++++) <100; (+++) 100 to 500; (++) 501 to 1000; (+) >1001 to 2000; (−) >2001. Strain of C. *dubliniensis* = *C. dubliniensis*; strain of *C. glabrata* *=* *C. glabrata*^2^; strain of *C. krusei* *=* *C. krusei*^3^; strain of *C. parapsilosis* = *C. parapsilosis*^4^; strain of *C. tropicalis* = *C. tropicalis*^5^.

**Table 5:** In vitro antifungal activity of phytoconstituents isolated from essential oils against *Candida* spp. strains.

Plant species	Source	Microorganism	MIC (*μ*g/mL)	MFC (*μ*g/mL)	Score MIC	Ref.
Citral	Commercial source	*Candida albicans* ATCC 76645	32	32	++++	[34]
		Clinical isolates *Candida albicans*	32–64	32–64	++++	

Linalol	Commercial source	*C. albicans* CA 032	2000	2000	+	[35]
		*Candida albicans* 051	1000	2000	+	
		Clinical isolate *Candida tropicalis*	500	500	+++	
		Clinical isolates *Candida krusei*	2000	2000	+	

*α*-Pinene	Leaves	*Candida albicans* clinical strain	500	>2001	++	[21]

Terpinen-4-ol	Commercial source	Clinical isolates *C. albicans*	>2001	nt	−	[31]

*γ*-Terpinene	Leaves	*Candida albicans* clinical strain	>2001	>2001	−	[21]

Thymol	Commercial source	*C. albicans* CBS 562	39	39	++++	[36]
		*C. tropicalis* CBS 94	78	78	++++	
		*C. krusei* CBS 573	39	39	++++	

Note: comparative MIC values (*μ*g/mL): (++++) <100; (+++) 100 to 500; (++) 501 to 1000; (+) >1001 to 2000; (−) >2001.

**Table 6:** Drug formulation from essential oils, study design, and outcomes of the randomized clinical trials included in this literature review.

Plant species	Essential oilformulation	Study design	Sample size	Country	Age (mean ± SD)/gender (Fem)^†^	Sample loss/reasons	Control group	Dosing protocol	Assessment check points	Assessment instruments of interest	Outcome	Ref.
*Pelargonium graveole ns*	Gel	Phase II, randomized, double-blind	80 patients (40 treated with Pelargonium gel and 40 treated with placebo)	Iran	38 to 78 years (61.39 ± 9.038)/(51 women and 29 men)	−	Treated with placebo (base gel 1% geranium essence)	Application of the gel twice a day (morning and night) for 14 days	2 weeks	Collection and culture of mycological samples from the palatal mucosa at each visit and colony count	+	[37]

*Zataria multiflora*	0.1% gel	Phase II double-blind, open randomized and controlled	24 patients (12 treated with miconazole gel and 12 treated with *Zataria multiflora* gel)	Iran	24 (15 women and 9 men) aged 45 to 83 years (average 60.83) years	−	Miconazole gel (2%)	The gel was applied to the base of the denture four times a day for 4 weeks	4 weeks	Colony counting of samples from the palatal mucosa, erythematous lesion on the palatal surface and from the surface of the denture	+/+	[38]

*Melaleuca alternifolia*	Alcoholic and nonalcoholic solutions	Phase II randomized, single-center open clinical trial	27 patients (13 treated with the alcoholic solution and 14 treated with the nonalcoholic solution)	USA	Men and women aged 18 to 65 years	5 (two did not return to receive the study medication and three received the medication but never returned for follow-up)	−	Group I: rinse using 15 mL of the solution for 30–60 s four times a day for 14 days; Group II: rinse using 5 mL of solution for 30–60 s, four times a day for 14 days	2 and 4 weeks	Assessment of signs and symptoms (thrush and erythema), extent of lesions, assessment of cure, improvement, change or worsening of oropharyngeal candidiasis	+	[39]

*Melaleuca alternifólia*	Cream	Phase II, randomized clinical trial	27 patients (3 groups of 9: control group, *Melaleuca artenifolia* group and , nystatin group)	Chile	50 to 77 years old/26 women and 1 man	−	Cream alone and cream + nystatin	For every 5 ml of cream, 1 ml was replaced by 1 ml of *M. alternifolia* cream and homogenized for 20 seconds	−	Culture and CFU count of samples from the palate mucosa in all sessions	+	[40]

*Melaleuca alternifólia*	Oral solution	Phase II, single-center open study	13 patients	USA	18 and 65 years old/men and women	1 (never returned)	−	Rinse using 15 ml of the solution for 30–60 s four times a day for 14 days	2 and 4 weeks	Assessment of signs and symptoms of oropharyngeal candidiasis, mycological assessments included a KOH test, yeast quantification, and in vitro susceptibility studies	+	[41]

Note: statistically significant reduction (+) or not (−) in the CFU count and in the signs and symptoms of oral candidiasis in relation to the positive control or placebo. ^†^Good result.
